# Light Increases Energy Transfer Efficiency in a Boreal Stream

**DOI:** 10.1371/journal.pone.0113675

**Published:** 2014-11-20

**Authors:** Jūratė Lesutienė, Elena Gorokhova, Daiva Stankevičienė, Eva Bergman, Larry Greenberg

**Affiliations:** 1 Marine Science and Technology Center, Klaipėda University, Klaipėda, Lithuania; 2 Department of Biology, Karlstad University, Karlstad, Sweden; 3 Department of Applied Environmental Sciences, Stockholm University, Stockholm, Sweden; 4 Institute of Ecology, Nature Research Centre, Vilnius, Lithuania; NERC Centre for Ecology & Hydrology, United Kingdom

## Abstract

Periphyton communities of a boreal stream were exposed to different light and nutrient levels to estimate energy transfer efficiency from primary to secondary producers using labeling with inorganic ^13^C. In a one-day field experiment, periphyton grown in fast-flow conditions and dominated by opportunistic green algae were exposed to light levels corresponding to sub-saturating (forest shade) and saturating (open stream section) irradiances, and to N and P nutrient additions. In a two-week laboratory experiment, periphyton grown in low-flow conditions and dominated by slowly growing diatoms were incubated under two sub-saturating light and nutrient enrichment levels as well as grazed and non-grazed conditions. Light had significant positive effect on ^13^C uptake by periphyton. In the field experiment, P addition had a positive effect on ^13^C uptake but only at sub-saturating light levels, whereas in the laboratory experiment nutrient additions had no effect on the periphyton biomass, ^13^C uptake, biovolume and community composition. In the laboratory experiment, the grazer (caddisfly) effect on periphyton biomass specific ^13^C uptake and nutrient content was much stronger than the effects of light and nutrients. In particular, grazers significantly reduced periphyton biomass and increased biomass specific ^13^C uptake and C:nutrient ratios. The energy transfer efficiency, estimated as a ratio between ^13^C uptake by caddisfly and periphyton, was positively affected by light conditions, whereas the nutrient effect was not significant. We suggest that the observed effects on energy transfer were related to the increased diet contribution of highly palatable green algae, stimulated by higher light levels. Also, high heterotrophic microbial activity under low light levels would facilitate energy loss through respiration and decrease overall trophic transfer efficiency. These findings suggest that even a small increase in light intensity could result in community-wide effects on periphyton in boreal streams, with a subsequent increase in energy transfer and system productivity.

## Introduction

According to stream ecosystem theory, riparian vegetation has the potential to have strong system-level effects on stream biota, through its organic inputs and physical influences [1]. In pristine headwaters, riparian shade can reduce light penetration and hence production of autochthonous algae [2]–[4]. However, even in shaded streams with low algal densities, primary production is efficiently incorporated into secondary production [5]–[7] because of the high digestibility and nutritional value of algal biomass as compared to nutrient-poor terrestrial detritus [7], particularly terrestrial detritus derived from coniferous trees [8].

Anthropogenic processes have a high potential to alter the light and nutrient regimes in boreal streams. Deforestation of catchments leads to increases in terrestrial inputs of dissolved and particulate organic matter in streams and reduces watershed retention of deposited nitrogen [9], [10]. The increased nutrient inputs to the streams could stimulate algal growth; however, suspended particulate matter and water coloration due to dissolved organic carbon may limit light for photosynthesis [11]. Moreover, increased atmospheric deposition of nitrogen has been suggested to induce a large-scale gradient in N:P ratios, with nitrogen limitation in the northern pristine areas gradually changing to phosphorus limitation in the industrial south of Scandinavia and presumably most of the Northern hemisphere [12]–[14]. Therefore, forestry effects on the stream periphyton are expected to vary geographically, depending on regional nutrient loading, watershed altitude, soil type, and various anthropogenic disturbances [15], [16]. Development of forest management tools, such as leaving riparian buffers [17], [18], requires knowledge of limiting factors for primary productivity and sensitivity of local periphytic communities to light and nutrient alterations in the streams, with effects of the rest of the stream food chain.

Light and dissolved nutrients have interactive effects on growth and nutrient content, i.e. nutritional value of primary producers in streams. The imbalance of nutrient stoichiometry in primary producers is regarded as an important factor limiting trophic transfer efficiency in aquatic food webs [19], [20]. Many studies emphasize light as a primary limiting factor for algal growth, whereas nutrients often become important only above saturating light levels [21]. However, nutrients have also been reported to limit algal growth even at irradiances below photosaturation (i.e. 100 µmol photons m^−2^ s^−2^) [22], [11], [23]. This implies that at low light levels, algae could efficiently retain nutrients and produce highly nutrient-rich organic matter, supporting secondary production in small headwater streams [24], [10], [25]. The light:nutrient hypothesis [27] predicts that high light conditions enhance photosynthesis and carbon uptake, resulting in production of nutrient (phosphorus) deficient organic matter. Therefore, increased light levels should lead to increased algal C:P ratios and decreased food quality for grazers, because P supply is essential for maintaining high growth rates of consumers [27]. High C:P biofilms on rock surfaces, with subsequent limited secondary production, are commonly observed under high light conditions if nutrient availability for primary producers is relatively low as commonly observed in oligotrophic lakes and streams [28], [29].

The possible limitation of nutrients is difficult to detect, because of the weaker effect of nutrients as compared to that of light and long incubation time required to detect nutrient addition effects in low light systems [30], [25], [11]. Moreover, small streams have low storage capacities of rainfall water in their watersheds, especially after forest removal, and undergo frequent changes in stream flow [16]. Water level can decrease rapidly, leaving most of the stream bed exposed to the air. Such conditions are advantageous for opportunistic algal species that quickly colonize the exposed surfaces [16]. However, little is known about the nutrient demands of these opportunistic algal communities and their importance for production in oligotrophic streams, partly because changes in biomass and chlorophyll *a* are difficult to detect in short-time growth experiments.

Using inorganic carbon labeled with ^13^C as a tracer of C incorporation, we evaluated the combined effects of different light and nutrient levels on the growth of benthic algae in a humic, oligotrophic boreal forest stream located in southern Sweden. Combining a one-day field and a two-week laboratory experiments, we tested the following hypotheses: (1) below saturating irradiances, photosynthetic carbon uptake in periphyton is positively related to nutrient enrichment; (2) light-nutrient interactions affect nutrient ratios (C:N, C:P) in periphyton, with nutrient deficient (high C:nutrient) organic matter produced under higher light; and (3) this nutrient-deficiency reduces the trophic transfer efficiency of nutrients from primary producers to grazers. Grazers may physically affect biofilm density and thickness, thus improving nutrient penetration into periphyton matrices, with subsequent effects on competition for light and nutrients among periphytic algae [31]. Moreover, invertebrates modify nutrient availability by excretion [32]–[34], physical perturbation of periphyton mats, and reducing boundary layer thickness [35]. In indoor laboratory tanks, we manipulated grazer abundance and observed how ^13^C uptake and nutrient ratios in periphyton are modulated by grazers. We also evaluated light and nutrient effects on energy transfer efficiency from primary to secondary producers by measuring the ^13^C signal in grazers.

## Materials and Methods

### Ethics Statement

No permits were required for sampling the streams, nor did we need ethical approval for working with invertebrates. Moreover, none of the species in the stream were endangered.

### Study site

**Figure 1:**
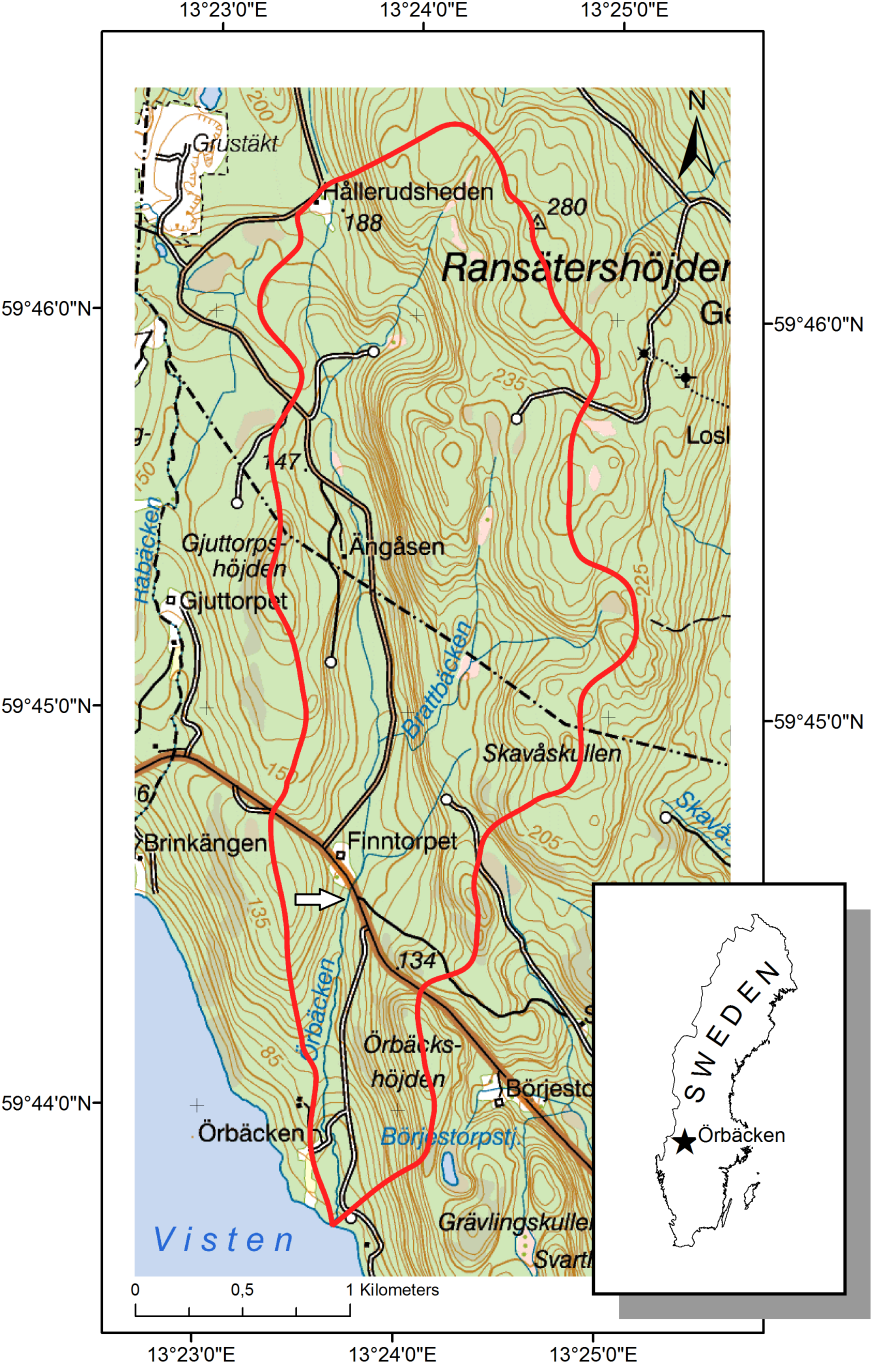
The study site as indicated by the arrow in Örbäcken Creek, western Sweden. The red line shows the catchment area (5.38 km^2^).

Two experiments were conducted: a field incubation experiment in the stream Örbäcken Creek, and a laboratory experiment using water and substrate material from the same stream. Örbäcken Creek, situated in western Sweden (N 59°44.493; E 13°23.698; Figure 1), is a third order stream, 1–1.5 m width, with cobbles and boulders as the dominant bottom substrate at the study site. The landscape is hilly, with elevation ranging from 196 to 370 m above sea level (a.s.l.), averaging ∼200 m a.s.l. [36]. Forestry activities in the watershed include selective forest cutting and clear-cutting. Canopy cover above the stream was ∼80–100%, with Northern spruce (*Picea abies*) being the dominant tree species. Depth and velocity of the stream varied substantially due to precipitation: the field experiment was conducted under medium flow conditions (25–26 May 2010), whereas material for the laboratory experiment was collected at low flow (9 June 2010). Decreased flow appeared to be related to changes in periphyton structure: mats of filamentous green algae dominated the periphyton community in May (pers. observations), whereas algal communities were dominated by loosely attached diatoms in June. Algal succession together with increased deposition of suspended solids determined initial differences in substrate used for the field and laboratory experiments (Table 1). Physicochemical parameters in the water during the period May 25–June 9 were as follows: pH 5.8–6.3, conductivity 22 µs cm^−1^, and temperature 11.7–14°C. Stream water contained humic substances (0.12–0.16 OD units measured as absorbance at 455 nm), which is typical for boreal Fenno-Scandia [37].

**Table 1:** Main characteristics and stoichiometry of the periphyton used in the field and laboratory experiments; (mean ± SE, AFDW = ash free dry weight, TSM = total sedimentary matter).

Variables	Field experiment(n = 4)	Laboratoryexperiment (n = 3)	t-test, p value
Chlorophyll a, µg cm^−2^	1.6±0.22	0.71±0.25	p<0.05
AFDW, mg cm^−2^,	1.02±0.19	0.51±0.07	n.s.
TSM, mg cm^−2^	5.43±0.69	5.69±1.13	n.s
Relative contribution oforganic matter to TSM, %	19±2	9±1	p<0.05
Autotrophic Index	634±75	836±165	n.s.
C, µg cm^−2^	357±50	245±68	n.s.
N, µg cm^−2^	37.8±5.4	12.4±2.7	p<0.05
P, µg cm^−2^	2.1±0.4	1.8±0.3	n.s.
C:N molar	11±0	11±1	n.s.
C:P molar	463±59	178±49	p<0.05
N:P molar	41.8±4.6	16.3±3.9	p<0.05
C:Chlorophyll *a*	227±28	179±32	n.s.
δ^13^C ‰	–32.1±0.5	–28.8±0.1	p<0.05

Unpaired t-tests were used to compare specific parameters between the field and laboratory experiments, n.s. = not significant, n = number of replicates.

### Field Experiment

*In situ* periphyton incubation was performed to detect if the nutrients (nitrogen and/or phosphorus) were limiting. In this experiment, a 2×4 factorial (2 light levels×4 nutrient treatments) design was applied, using periphyton on stones from the forested stream section incubated for 24 h under static water movement conditions. The experiment was conducted in 32 open (vertically oriented) PVC containers (volume 14 L), submerged in two 100 m stream sections: a closed-canopy forested and open stream section (Figure S1 in Supplementary material). We acknowledge that this light manipulation cannot capture the total variation of environmental conditions related to light regime in forest streams; however, this simplification is both ecologically relevant and served well the hypothesis testing.

The experiment consisted of four treatments, each treatment being replicated four times. At each site, 4 nutrient addition treatments (P, N, N + P, and control - no additions) were applied. The additions represented enriched nutrient concentrations, corresponding to an approximately 2-fold increase in PO_4_-P and a 7-fold increase in NO_3_-N, as observed in nearby oligotrophic headwater streams during the growth season (Table 2). The increased N:P ratio, which drives the system towards phosphorus limitation is ecologically relevant in this region, as there is anthropogenically-induced atmospheric deposition of N but not P [12]. Nutrient additions were accomplished by adding dissolved 0.3 µmol/l PO_4_-P as K_2_HPO_4_×3H_2_O and 10 µmol/l of NO_3_-N as KNO_3_. In both experiments, the final enriched N:P ratios were close to a N:P ratio of 18, which is optimal for benthic algae [38].

**Table 2:** Nutrient concentrations (µmol L^−1^) in the stream water during the field and laboratory experiments, and in the region during the summer (Nyberg L. pers. communication).

	Concentrations, µmol L^−1^				
	*in* *situ* experiment		Laboratory experiment		Region
Nutrient	Ambient	Enriched	Ambient	Enriched	Min-Max, Mean
PO_4_-P	0.26	0.56	0.35	0.65	<0.32
NO_3_-N	1.57	11.57	1.21	11.21	0–8.4, 1.4
NO_2_-N	0.57	0.57	0.21	0.21	n.d.
NH_4_-N	<0.71	<0.71	0.71	0.71	0–4.8, 0.2
N:P	10	22	6	19	>5

N.d. = no data.

To estimate the incorporation rate of ^13^C-labelled inorganic carbon by the periphyton community as a proxy for algal production rate, sodium bicarbonate NaH^13^CO_3_ (Cambridge isotopes, 99% heavy isotope) was added to all incubations to a final concentration of 0.02 mM. The experiment started in the afternoon and was terminated 24 h later; the water in the containers was mixed every 4 h. Physicochemical water parameters and PAR (a light meter LI-18, Leiderdorp Instruments) in the containers were measured at 19∶00 h and at 11∶00 h and 14∶00 h the following day (Table S1). PAR was significantly lower in the forested section than in the open section, both in the evening (19∶00 h; t test, p<0.05) and during the day (11∶00 h; non parametric median test, p<0.05; Table S1), with maximum values (1075 µmol m^−2^ s^−2^) recorded in the open habitat at 14∶00 p.m. Water temperature was significantly higher (1–1.6°C) in the open than in the forested sections at 19∶00 and 14∶00 (t test, p<0.05, in both cases), but was similar at 11∶00 (t test, p>0.05). There was a small decrease in conductivity from 23.1 to 22.9 µs cm^−1^ (paired t test, p<0.05), whereas pH remained constant during the one day of incubation (t test, p>0.05; Table S1).

### Laboratory Experiment

To investigate the effect of light, nutrients and grazers on periphytic algal communities, we conducted a 2-week long laboratory experiment in tanks with recirculating water. A split plot design was used [39], where experimental tanks were the plots, light (low and high) and nutrients (enriched and ambient) were the between plot factors, and grazers (caddisflies *Potamophylax* sp., Trichoptera) were the within-plot factor (present/absent). Naturally growing periphyton on stones (diameter 11±3 cm) from the stream was incubated in the tanks to monitor changes after 2, 5 and 14 days. The stones were taken from a slow-flowing, depositional area in the forested section of the stream. They were placed into plastic boxes, filled with stream water and transported to the laboratory within 2 h. Three stones were used per replicate tank. On each sampling occasion (day 2, 5 and 14), one stone was removed and periphyton was sampled for nutrients, isotope analyses, chlorophyll *a*, AFDW (ash-free dry weight) and community structure.

**Figure 2:**
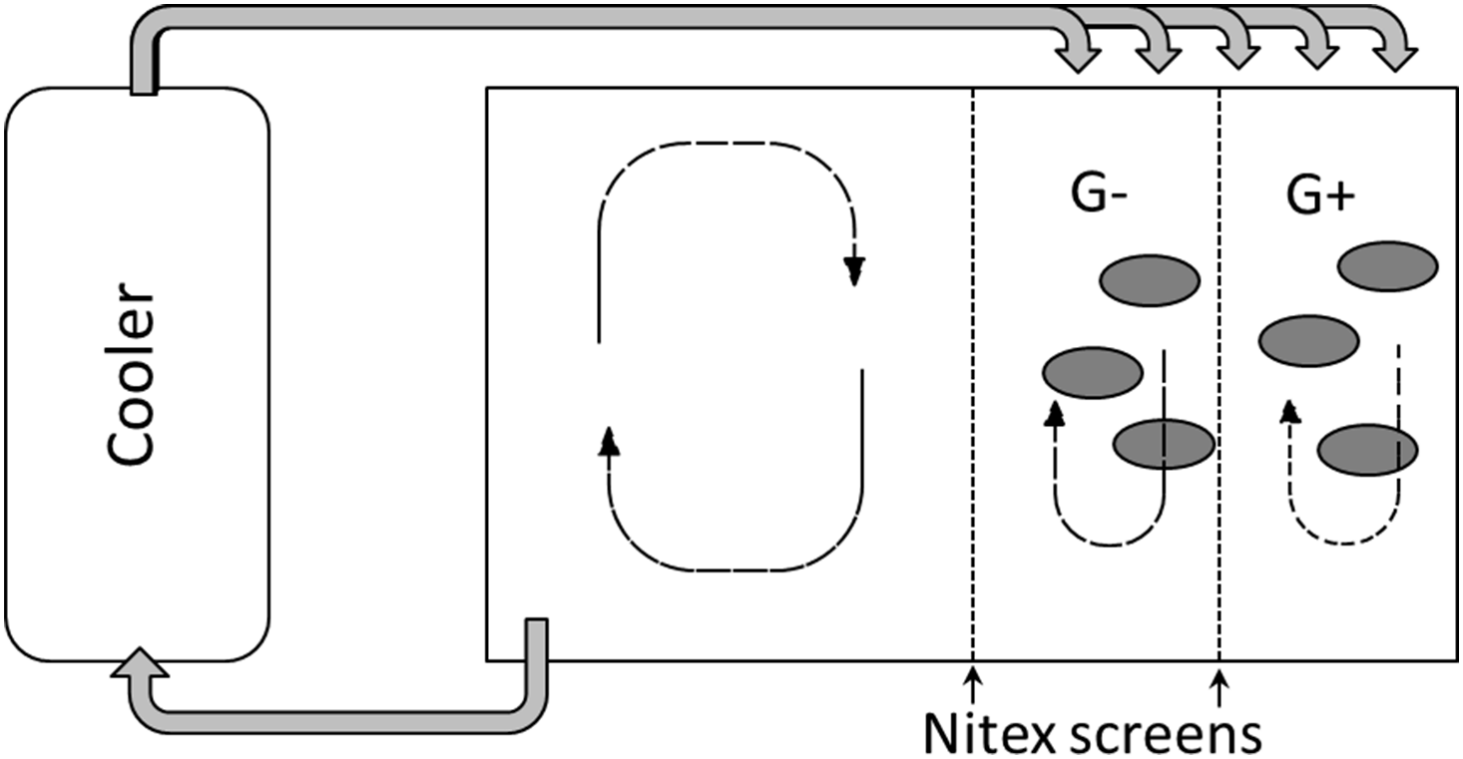
Schematic drawing of the experimental incubation system in the flow-through aquaria. Water was cooled by circulating it through the cooler as indicated by the arrows. Ellipses denote stones, and G+ and G− denote the addition or lack of grazers.

Twelve 200-L experimental tanks (100×50×40 cm) were used, giving 3 replicates for each treatment. Each tank was filled with 52 L of stream water, which was pumped through an automatic cooling system (TECO RA200/680), maintaining the water at 14°C, which was the ambient temperature in the stream. The water circulation created a turbulent flow of <0.2 m s^−1^, and depth was 11.4±0.2 cm. Each aquarium was divided into two sections; one section was further divided with Nitex net (1 mm mesh), producing two 25×50 cm subsections, with and without the caddisflies as grazers. The other half of the aquarium was left free for water circulation (Figure 2). The caddisflies (body weight 16.1±3.9 mgDW) were collected 3 days before the experiment to acclimatize them to laboratory conditions, and placed, at random, into one of the two sections of each aquarium (Figure 2). Six individuals were added to each section at the start of the experiment, and two individuals and one stone were removed on each sampling occasion to keep the density of caddisflies per stone constant. Caddisfly density in the experimental enclosures was 214±18 ind. m^−2^, similar to the density in Örbäcken Creek (personal observations). No mortality or metamorphosing occurred after 2 and 5 days, whereas 20% of the caddisflies metamorphosed to pupa after two weeks. As metamorphosing may induce changes in isotopic signal [40], only individuals after 5 days of incubation were taken for the SIA (Stable Isotope Analysis) and C transfer efficiency measurements. We were not able to remove all small-sized grazers, such as chironomids and chydorids, when we brought the stones into the laboratory. However, their observed densities were low and their influence was considered negligible.

Two light levels, 6±2 and 40±4 µmol m^−2^ s^−2^ (PAR) were created by combining halogen and warm light sources. The difference in light levels simulates the change in irradiance after forest thinning. A photoperiod of 14 h light and 10 h of darkness, with 30-min of artificial sunset and sunrise, was maintained.

Nutrient enrichment of both PO_4_-P and NO_3_-N was done at the same concentrations as in the field experiment, and thus enriched concentrations were similar in the two experiments (Table 2). Sodium bicarbonate NaH^13^CO_3_ was added to a final concentration of 0.04 mM. A higher concentration of NaH^13^CO_3_ was used in the laboratory experiment than the field experiment to adjust for the expected isotopic signal to the lower densities of periphytic algae observed in the laboratory experiment (Table 1). Conductivity, pH and absorbance as a proxy for humic substances (measured at 455 nm after filtration through the GF/C filter with 1.2 µm pore size) were measured on each sampling occasion. There was small but significant decrease in water absorbance and increase in pH over time (RM ANOVA, time effect: p<0.05, Table S1), whereas there were no significant effects of light and nutrient treatments or related interactions with time. Conductivity remained constant throughout the experiment and among treatments (RM ANOVA, time effect: p>0.05, light and nutrient effect: p>0.05).

### Sample preparation and analysis

Sampling of periphyton involved placement of a 5.5 cm diameter (23.75 cm^2^ area) plastic lid on the periphyton-covered surface of the stone and cleaning the surrounding area with a brush. The remaining periphyton beneath the lid was then brushed, collected with pre-filtered stream water into 100 ml plastic beakers and stored cool during transportation. The suspension was mixed and a syringe was used to take 10–40 ml subsamples for filtering and Lugol fixation. Pre-combusted (for 2 h at 520°C) and pre-weighed (±0.0002 g) 25 mm diameter Whatmann GF/C filters were used to concentrate samples for SIA, and 47 mm filters were used to collect samples for chlorophyll *a* and AFDW measurements. For the chlorophyll *a* analysis, the filters were frozen at −20°C. Chlorophyll *a* was measured after extraction in 90% methanol and 5 min sonication in an ultrasonic bath, according to Simon and Helliwell [41]. The dry weight of total sedimentary matter (TSM) was estimated by drying the filters at 105°C to a constant weight. The same filters were then reweighed after 2 h of combustion at 520°C to estimate AFDW. An Autotrophic Index (AI) was calculated as the ratio of AFDW:chlorophyll *a* according to Bigs and Close [42]. Increased ratio indicate that heterotrophs utlizing organic substances comprise a larger percentage of AFDM than autotrophic periphyton that rely largely on inorganic nutrients to increase biomass.

Samples for SIA and elemental (C, N, and P) analysis were dried on GF/C filters for 48 h at 60°C and weighed. Half of the filter was packed into a tin capsule for SIA and CN analysis. Caddisflies were dried, grinded to fine powder in agate mortar and weighed in the tin capsules for SIA. Ratios of ^13^C/^12^C as well as %C and %N in the samples were determined using continuous-flow isotope mass spectrometry provided by Automated NC Analysis (ANCA) SL 20-20, PDZ Europa at the Stable Isotope Facility, UC Davis, USA. The standard reference materials were Vienna PDB and atmospheric N_2_. Isotope ratios were expressed as parts-per-thousand (‰) differences from the standard reference material [43]. The other half of the filter was used for analysis of total phosphorus that was measured using the molybdate-ascorbic acid method, after potassium persulfate digestion at 120°C for 30 min. Samples were centrifuged (at 4000 g for 10 min) before they were analysed spectrophotometrically as in Færøvig and Hessen [44].

Algal cells were counted in the Fuchs–Rosenthal counting chamber (volume 0.0032 ml) under a light microscope at ×600 magnification. The counting units were a 100 µm section of a filament length for filamentous species (cyanobacteria and algae), a colony or a coenobium for colonial and coenobian algae, and a cell for unicellular species. Species- or genus- specific counts were converted to biovolume by measuring the dimensions (in µm) of 10 cells of each taxon and using geometric equations [45].

### Data analysis and statistics

If not specified otherwise, the mean values and standard errors (SE) are given. We used δ^13^C values of periphyton as a proxy for photosynthetic assimilation rate by periphytic primary producers. The δ^13^C uptake by periphyton communities varies with relative contribution of algal and non-algal material; however it was impossible to separate these constituents in the samples used for SIA. We calculated algal biomass-specific ^13^C uptake by dividing the incorporated ^13^C by periphyton (denoted as Δ^13^C and calculated as a difference between the mean initial δ^13^C value and the value at the time of the observation) by the periphyton chlorophyll *a* content (µg cm^−2^) representing the autotrophic component. As a proxy of C transfer efficiency from primary to secondary producers, the Δ^13^C_caddisflies_/Δ^13^C_periphyton_ ratio was used.

For the field experiment data, Box-Cox transformed Δ^13^C values were used in a generalized linear model (GLM) to estimate nutrients (nitrogen and/or phosphorus), light and autotrophic periphyton thickness (as chlorophyll *a*) as well as molar ratios of C:nutrients as continuous predictors effects. The model fitting was performed using the Akaike information criterion procedure.

Repeated measures two factorial ANOVAs (RM ANOVA) or non-parametric Friedman tests were performed to test for temporal changes in water chemistry during the laboratory experiment. Logarithm or cube root transformations were used to achieve normality and homogeneity of variances. To test for light and nutrient effects on periphyton (Box-Cox transformed) parameters, RM ANOVA was applied with two within-subject (repeated measures) factors: grazing (grazed vs. ungrazed communities) and sampling time (day 2, 5 and 14). The following null hypotheses for grazer (time) effects were tested: (1) there are no differences in the parameter (^13^C uptake, nutrient ratios, etc.) between grazing (time) levels; and (2) the effects of light, nutrient or light×nutrient were the same at all grazing (time) levels.

## Results

### Field experiment: ^13^C uptake

**Figure 3:**
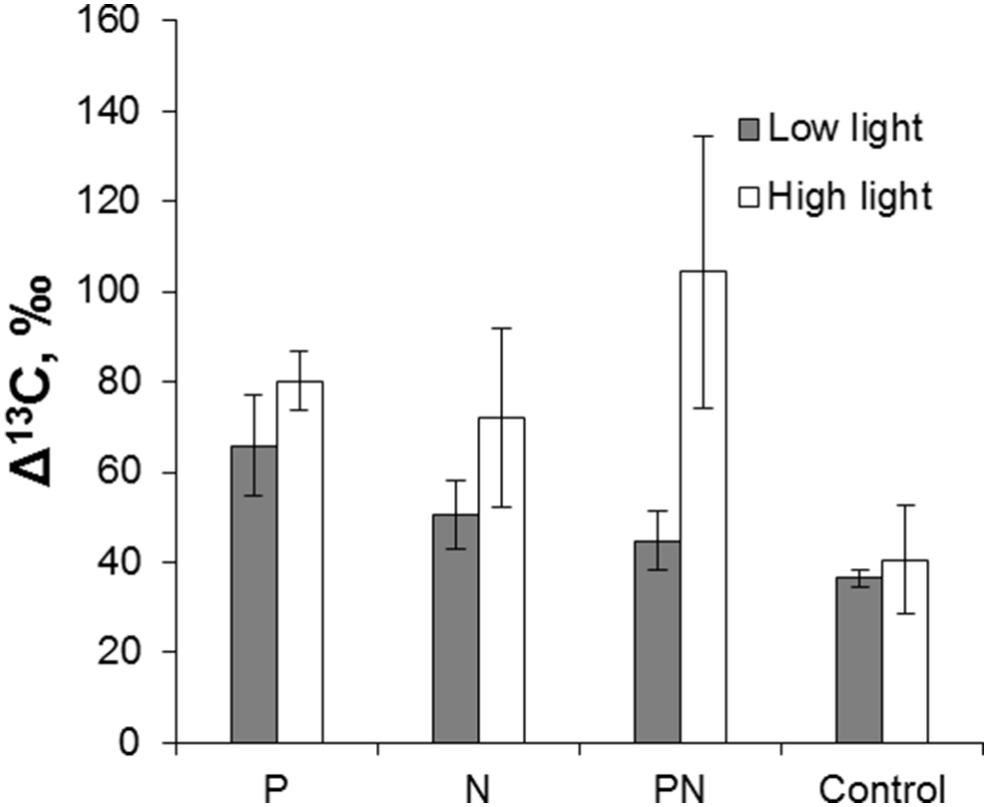
Mean values (± SE) of the δ^13^C increment (Δ^13^C) in periphyton grown under different light and nutrient (P- phosphorus, N- nitrogen) additions in the field experiment.

No significant effects of light and nutrients on chlorophyll *a* and AFDW in periphyton were observed (two-way ANOVA’s, p>0.05). In all incubations, the ^13^C enrichment increased significantly from the initial −32.1 ‰, with an average Δ^13^C of 62±34 ‰ (Figure 3, Table S2). The Δ^13^C values showed positive response to both light and P addition (Table 3). The ^13^C uptake by periphyton was negatively related to both C:N ratio and chlorophyll *a*, with a 5-fold greater effect size for the latter. Nitrogen addition, C:P and N:P caused no significant effect on Δ^13^C.

**Table 3:** The GLM of Box-Cox transformed Δ^13^C values in periphyton from the field experiment.

Variable	Level of effect	Estimate	Wald	p
LnChlorophyll *a*		–0.2	12.15	0.0005
C:N		–0.04	5.58	0.0182
Light	LL	–0.05	12.77	0.0003
P	not added	–0.05	9.94	0.0016

Light (high vs. low, LL) and P – phosphorus (added vs. not added) are categorical factors, whereas chlorophyll *a* (µg cm^−2^) and C:N (molar) are continuous predictors.

### Laboratory experiment

#### Algal community responses to light, nutrient and grazer effects

**Figure 4:**
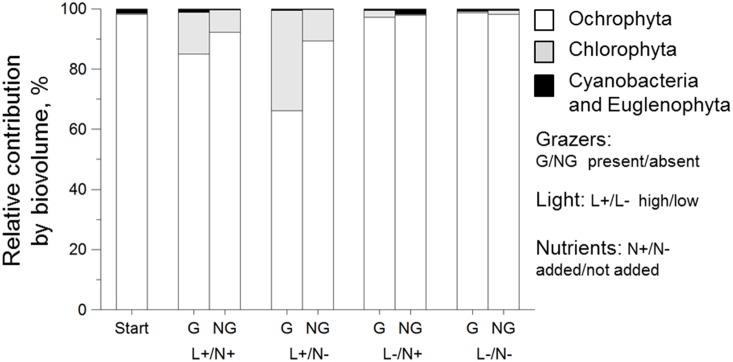
Composition of periphytic algae. % of total biovolume at the start and at the end of the laboratory experiment for grazed and ungrazed periphyton at the two light levels: (L+ = PAR of 40 µmol m^−2^ s^−2^ and L– = PAR of 6 µmol m^−2^ s^−2^). N+ indicates nutrient enrichment and N– ambient nutrient levels. White bars represent diatoms, grey are green algae and black are cyanobacteria and euglenophytes.

**Figure 5:**
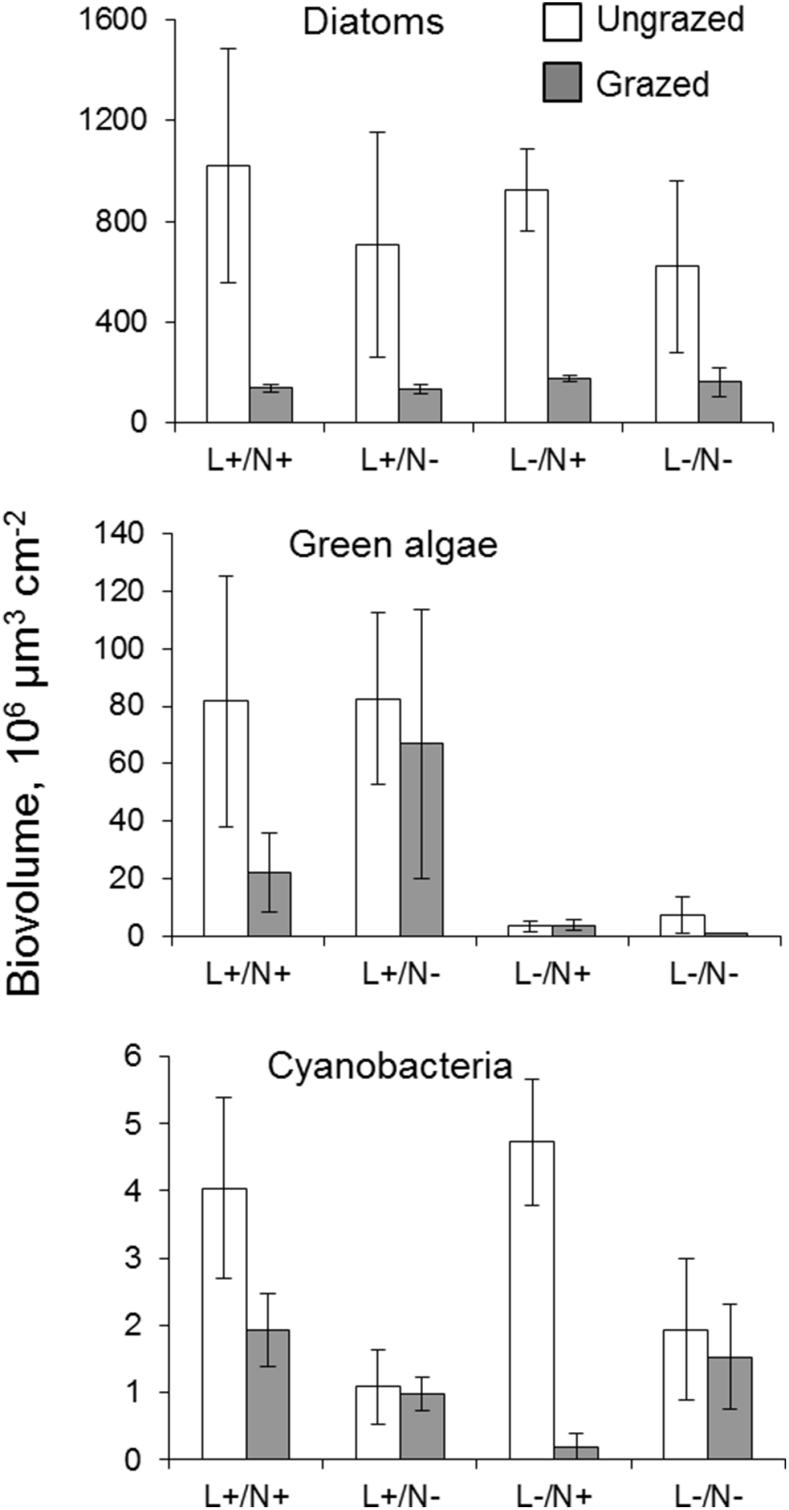
Algal biovolume. Mean (± SE) biovolume of diatoms, green algae and cyanobacteria subjected to grazing/non-grazing and four different light (high = L+; low = L–) and nutrient (addition = N+; ambient = N–) conditions at the end of the laboratory experiment. Note the scale of the y-axis differs among panels.

At the start of the laboratory experiment, *Navicula* contributed 82–87%, *Eunotia* – 9–15%, whereas other diatoms (*Achnanthes*, *Nitzschia* and *Denticula*), filamentous cyanobacteria and green algae (*Mougeotia* sp. and *Monoraphidium* sp.) comprised <1% of the total biovolume (Figure 4). After two weeks of incubation in the laboratory, the green algae *Ulothrix*, *Closterium* and *Scenedesmus* comprised a large portion of the algal community (Figure 4, Table S3). The diatom assemblage was dominated by the same taxa as at the start of the experiment but with small contributions from several new taxa, such as *Tabellaria*, *Fragilaria* and *Pinnularia*. Diatoms were most affected by grazers, but showed no response to light and nutrient manipulations (RM ANOVA, grazer effect: p<0.05; light effect: p>0.05; nutrient addition effect: p>0.05, Figure 5). Green algae responded positively to the light increase at both grazer levels (RM ANOVA, light effect: p<0.05, grazer: p>0.05), whereas cyanobacteria responded positively to nutrient addition only in the non-grazed treatments (RM ANOVA, nutrient×grazer interaction: p<0.05, two-way ANOVA for grazed periphyton, nutrient effect: p<0.05).

#### Periphyton biomass and C:N:P stoichiometry

**Figure 6:**
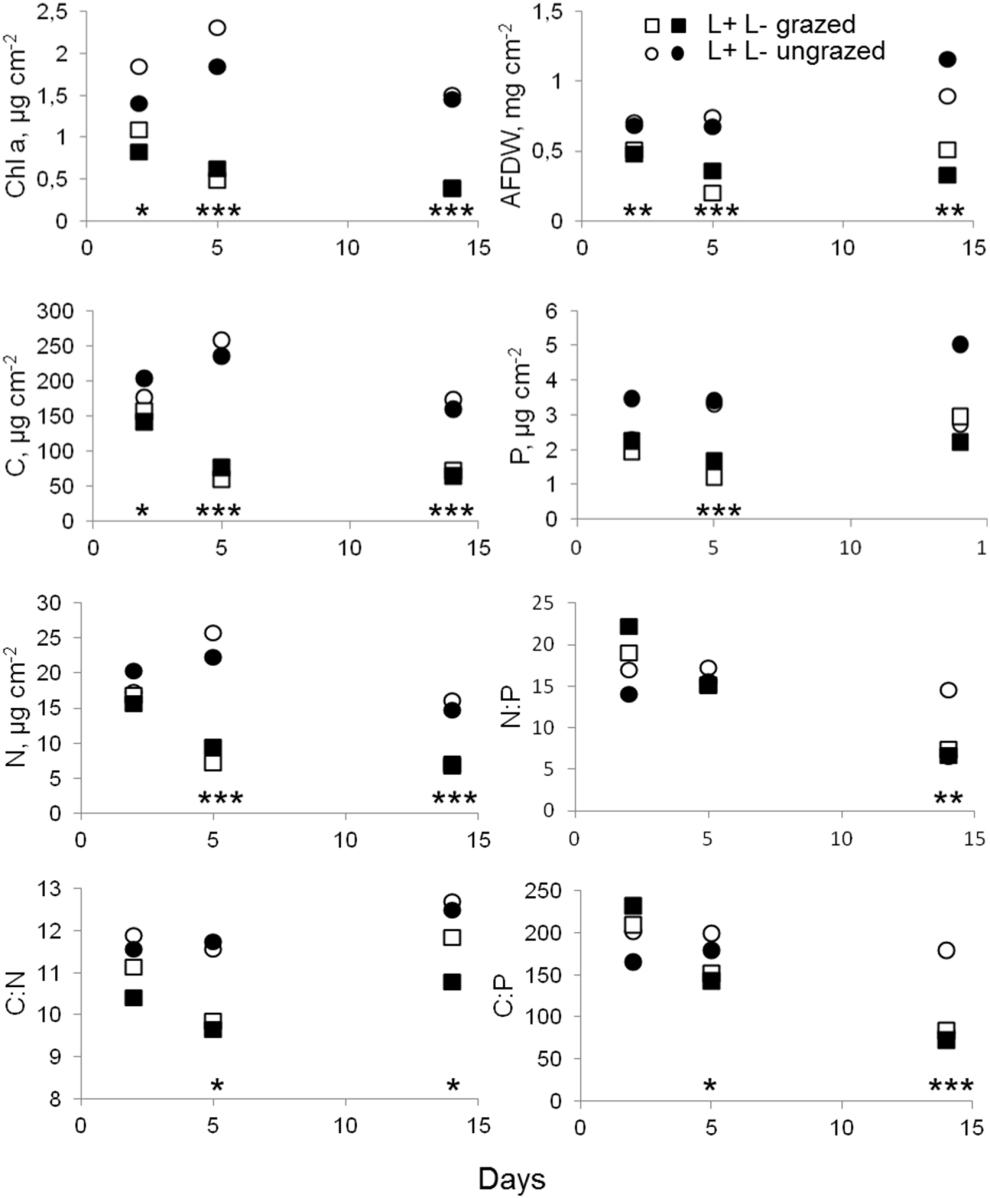
Mean values of periphyton biomass (chlorophyll *a* and AFDW), elemental (C, P, and N) concentrations and nutrient ratios (N:P, C:N, and C:P) over the 14 day-long laboratory experiment. Squares and circles denote grazed and ungrazed conditions, respectively. Filled symbols denote low light and open high light treatments. Asterisks denote significant grazer effects for each sampling day (RM ANOVA, with grazing as within-group factor, *p<0.05, **p<0.01, ***p<0.001).

No significant effects of light and nutrients on chlorophyll *a* and AFDW were observed in the laboratory experiment (Table 4 and S4). Grazers reduced chlorophyll *a* and AFDW significantly; moreover, grazer effect increased with time (RM ANOVA time×grazing effects on AFDW and chlorophyll *a*: p<0.05 in both cases; Tables 4 and S5, Figure 6). In turn, nutrient ratios in periphyton changed significantly with time, but there were no significant effects of light and nutrients (Table 4, Figure 6). Grazers significantly reduced C, P and N content in periphyton (Figure 6), whereas the effect on the nutrient ratios was time-dependent, with significant negative effects on days 5 and 14 (RM ANOVAs, grazer effect: p<0.05, Table S5, Figure 6).

**Table 4:** Effect sizes (η^2^) for the effects of nutrient additions, light, grazing and time of sampling on periphyton biomass (chlorophyll *a* and AFDW), inorganic carbon uptake (Δ^13^C, biomass-specific ^13^C uptake (Δ^13^C/Chl *a* and Δ^13^C/AFDW), nutrient content (C, N, and P) and nutrient ratios (N:P, C:P, and C:N).

Parameter	Chl *a*	AFDW	Δ^13^C	Δ^13^C/Chl *a*	Δ^13^C/AFDW	C	N	P	N:P	C:P	C:N
Light	0.17	0.004	0.62**	0.41*	**0.32** ^0.09^	0.04	0.00	0.00	0.09	0.16	0.12
Nutrients	0.20	0.10	0.03	0.14	0.03	0.12	0.20	0.19	0.00	0.00	0.06
Light×nutrients	0.06	0.06	0.02	0.01	0.002	0.01	0.00	0.01	0.02	0.01	0.05
Time	0.18	0.32*	0.80***	0.10	0.23	0.60***	0.61***	0.45**	0.63***	0.64***	0.43*
Time×light	0.03	0.02	0.08	0.06	0.17	0.13	0.07	0.03	0.09	0.10	0.05
Time×nutrients	0.01	0.02	0.03	0.05	0.07	0.08	0.05	0.04	0.02	0.04	0.04
Grazing	0.9***	0.93***	0.59**	0.82***	0.78***	0.95***	0.96***	0.93***	0.00	0.21	0.63**
Grazing×light	0.12	0.02	0.15	0.03	0.31	0.05	0.01	0.00	0.23	0.25	0.06
Grazing×nutrients	0.00	0.02	0.05	0.01	0.00	0.07	0.04	0.05	0.00	0.01	0.06
Time×grazing	0.32***	0.34*	0.12	0.14	0.26*	0.69***	0.67***	0.53***	0.44***	0.40*	0.12

All data are from the laboratory experiment and analyzed by repeated measures ANOVA. Significant effects are in boldface and denoted by *p<0.05, **p<0.01, ***p<0.001; for marginally significant effects, p values are given. The 3rd order interactions are not presented as none were significant.

N and P were differently related to autotrophic and total periphyton biomass. The correlation coefficient for the relationship between the N content and chlorophyll *a* in periphyton (r = 0.77) was higher than for that between the N content and AFDW (r = 0.52). For P content, the situation was just the opposite, with a higher correlation between the P content and AFDW (r = 0.65) than between the P content and chlorophyll *a* (r = 0.35, p<0.05 in all cases). There was a significant trend of increasing AFDW with time, however without an increase in chlorophyll *a* (Figure 6). This resulted in a significant increase in AI with time (RM ANOVA, light, nutrient, grazer effect: p>0.05, time effect p<0.05, Table S4), implying dominance of heterotrophic periphyton components and some accumulation of non-algal organic matter during the course of the experiment. Assuming 0.1 conversion factor of algal wet mass to carbon [46], we estimated that at the start of the experiment the contribution of algal carbon was 87±10% of the total carbon. After 2 weeks of incubation, algal carbon decreased to 31±3% and 55±7% in the grazed and ungrazed communities, respectively (RM ANOVA; light×nutrient effects: p>0.05, grazing effect: p = 0.08).

#### ^13^C uptake in periphyton and transfer in the food chain

**Figure 7:**
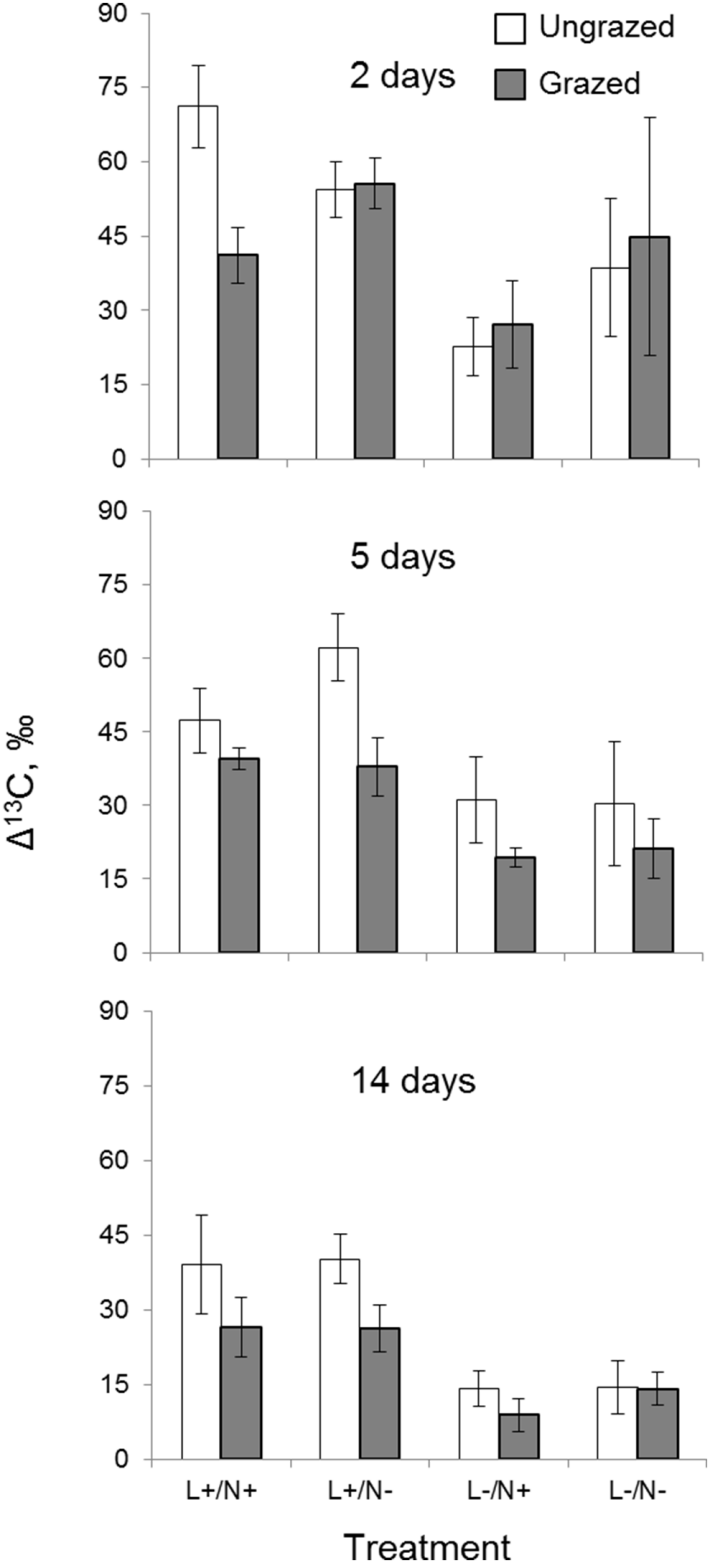
The δ^13^C increment (Δ^13^C) (means ± SE) in periphyton subjected to grazing/non-grazing and four different light (high = L+; low = L–) and nutrient (addition = N+; ambient = N–) conditions after 2, 5 and 14 days.

The δ^13^C increased significantly from an initial value of −28.8 ‰, with an average Δ^13^C of 44±22 ‰ after 2 days of incubation, followed by a gradual decline thereafter due to community respiration. ^13^C uptake was significantly higher at high light levels than at low light levels, whereas the response to nutrient addition was not significant (Table 4, Figure 7).

**Figure 8:**
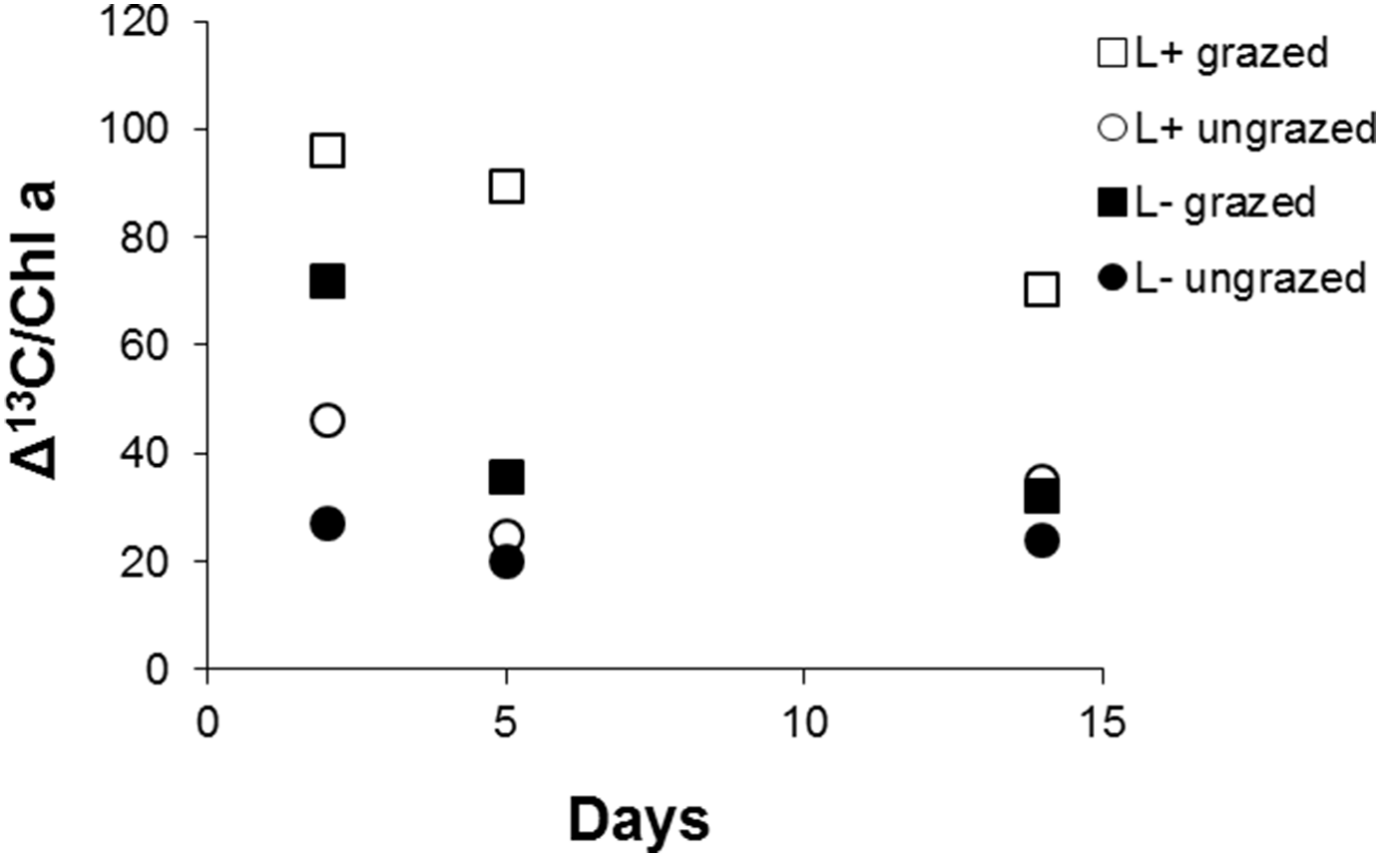
Mean biomass-specific ^13^C uptake for the four light and grazer treatments over time in the laboratory experiment. Squares and circles denote grazed and ungrazed conditions, respectively. Filled and open symbols denote low and high light treatments, respectively.

**Figure 9:**
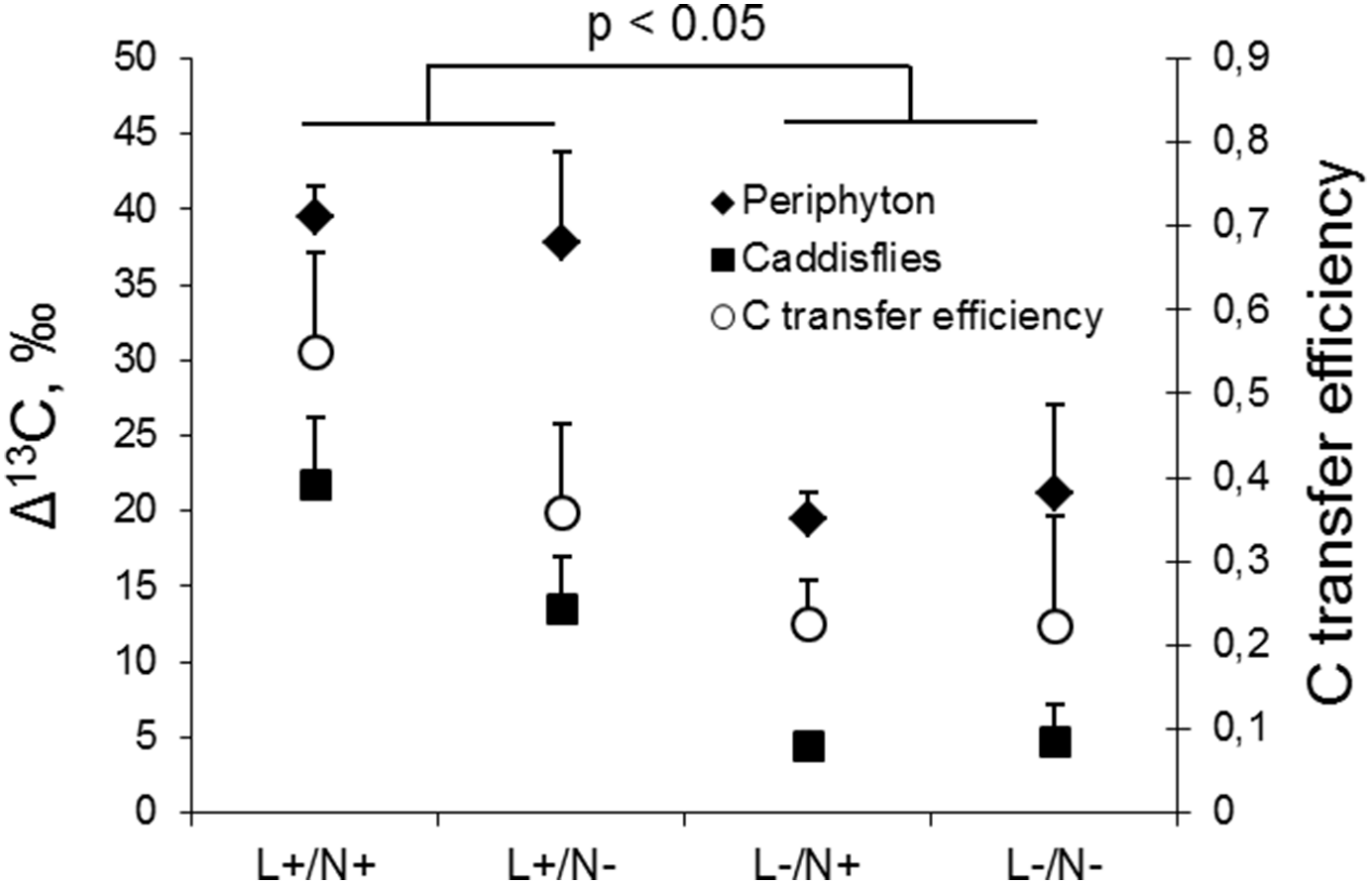
The δ^13^C increment (mean± SE) for caddisflies and periphyton, as well as the carbon transfer efficiency (expressed as Δ^13^C_caddisflies_/Δ^13^C_periphyton_) for the four different light (high = L+; low = L–) and nutrient (addition = N+; ambient = N–) conditions after 5 days in the laboratory experiment. The three separate two-way ANOVAs were used to test significance of light, the δ^13^C increment for caddisflies, periphyton and transfer efficiency (Δ^13^C_caddisflies_/Δ^13^C_periphyton_).

Grazers significantly reduced δ^13^C values in periphyton (Table 4, Figure 7). Moreover, there was a significant positive grazing effect on biomass-specific ^13^C uptake measured as Δ^13^C/chlorophyll *a* (RM ANOVA, grazing effect: p<0.001; Figure 8). The initial δ^13^C value of caddisflies was −28.7±0.4 ‰, which did not differ significantly from the stream periphyton (t test, p>0.05), indicating reliance on this food source *in* *situ*. The ^13^C uptake by periphyton and its transfer to the grazers resulted in a significant δ^13^C enrichment in the caddisflies (Figure 9); moreover, there was a significant linear relationship between Δ^13^C values in periphyton and caddisflies (r = 73, p<0.05). Under high light conditions, the grazers were most enriched in ^13^C, whereas nutrient addition caused no significant effect on Δ^13^C in caddisflies (two-way ANOVA, light effect: p<0.05). Similarly, the Δ^13^C_caddisflies_/Δ^13^C_periphyton_ ratio was positively affected by light, whereas the nutrient effect was not significant (two-way ANOVA, light effect: p<0.05, nutrient effect: p>0.05, Figure 9).

## Discussion

We found no support for the hypothesized positive effect of nutrient addition (hypothesis 1) on carbon uptake below saturated light levels in the laboratory experiment. However, there was a significant positive effect of phosphorus addition on periphyton growth (^13^C uptake) during the *in* *situ* incubations under forest light conditions. Also, we found no support for the predicted production of nutrient deficient (high C:nutrient ratio) organic matter as light levels increased (hypothesis 2). Finally, hypothesis 3, linking nutrient-deficiency to decreased trophic transfer, was not corroborated either, because trophic transfer efficiency was significantly limited by light as suggested by the positive effect of light on the Δ^13^C_caddisflies_/Δ^13^C_periphyton_ ratio, whereas nutrient addition had no effect.

### Light and nutrient effects

Our findings from the field experiment are consistent with previous studies of oligotrophic streams that report enhanced algal growth under P-addition, even in heavily shaded stream sections [23], [22]. One proposed mechanism for this phenomenon is the increased demand of nutrients for photoadaptation at low light levels [11]. There are several possible explanations for the lack of a nutrient addition effect in the laboratory experiment related to the low degree of nutrient limitation, initial differences in the periphyton communities and water flow conditions used in our studies. In the field incubations, we used a low-profile community developed under high flow conditions, dominated by tightly-packed, fast-growing filamentous green algae, whereas in the laboratory experiment, the periphyton community was largely dominated by loosely attached or motile taxa adapted to low flow conditions, mainly slow-growing diatoms. Architecture of the periphyton matrix influences nutrient advection through the mat, where water velocity determines the thickness of the benthic boundary layer around the matrix [47]. Static flow conditions in our *in* *situ* mesocosm enclosures increased boundary layer thickness and probably enhanced nutrient limitation and ^13^C access for algae, as demonstrated by the negative relationship between ^13^C uptake and chlorophyll *a*. As a consequence of lower periphyton matrix permeability in these communities, the stoichiometric imbalance between the surrounding media and the periphyton was more pronounced in the field than in the laboratory experiment. The nutrient ratios in the periphyton used for *in* *situ* incubation (N:P>32 and C:P>369) [38] indicate P limitation, although ambient nutrient concentrations in the water (N:P = 10, Table 2) implicate N as the limiting element rather than P. This result corroborates earlier findings that nutrient ratios in stream periphyton may be decoupled from the ratios in the water, implying that applicability of the Redfield ratio to infer the limiting nutrient is rather limited [48], [49]. Indeed, in the periphyton used for our laboratory experiment, N:P ratio was close to optimal for such communities (N:P = 18) [38], and there was no negative relationship between the ^13^C uptake and periphyton thickness, which indicates no restricted penetration of ^13^C and, presumably, nutrients into the periphyton matrix.

Communities dominated by slow growing diatoms were used for the laboratory experiment. Diatoms could be the most likely algal group to respond positively to nutrient additions as they are well adapted to low light conditions [50]. However, they are also likely to be more resistant to nutrient limitation than other algal species, because low nutrient concentrations and light intensities frequently co-occur in undisturbed forested streams [11]. Indeed, in our 2-week indoor experiment, the biovolume of diatoms tended to respond positively to nutrient enrichment, but the effects were not significant, which might indicate that longer time might be needed to detect changes in these slow-growing taxa. Cyanobacteria responded significantly to nutrient additions, but their contribution to community biovolume was too small (0.1–1.1%) to functionally influence community-level responses (chlorophyll *a*, or ^13^C uptake). By contrast, periphyton incubated at low light levels in the field showed enhanced ^13^C uptake when P was added. These findings suggest that opportunistic, fast-developing algal communities inhabiting temporarily submerged rock surfaces may require higher phosphorus levels to sustain their growth.

Another important issue that might have contributed to the different responses to nutrient additions at low light levels between the laboratory and the field settings is the significant proportion of detritus and heterotrophic organisms in the periphyton used in the laboratory experiment. We observed an increase in AI and phosphorus content in the periphyton during the course of the laboratory experiment that might be caused by heterotrophic production. Heterotrophic bacteria, flagellates and fungi associated with detritus may dominate phosphorus uptake in heterotrophic headwater streams [51], [52] and inhibit algal growth by competing for nutrients. Heterotrophic bacteria could also have masked some of the algal ^13^C uptake response in treatments with nutrient addition by assimilating ^13^C [53]. Our results support the view that in oligotrophic streams, algae may rely more on internal nutrient recycling rather than on streamwater nutrients [54], [49].

### Grazer effect

Caddisflies caused strong grazing pressure on the periphytic communities and removed ∼70% of biomass (as chlorophyll *a*) during the first 5 days of the incubation. Grazers also modified algal community structure and increased its diversity at high light levels. Increasing light (e. g., due to canopy removal) primarily stimulates growth of filamentous green algae in forest stream communities that are otherwise dominated by diatoms [17], [55]. Loosely attached taxa, such as diatoms, are more sensitive to grazers compared to prostrate or filamentous forms [25], [50]. Filamentous forms that dominated the green algae assemblage (e.g., *Mougeotia* and *Ulothrix*) in our study responded positively to the light increase, and their relative contribution was higher in the grazed than in the ungrazed treatments after 2-weeks, presumably due to negative selection on long filaments by grazers.

Grazers also increased the biomass-specific uptake of photosynthetic inorganic carbon. In periphyton, biomass-specific rates of productivity and nutrient uptake decrease with increasing biomass because of self-shading and reduced nutrient penetration into the microbial matrix [56]. The ^13^C stratigraphy in the periphyton matrix proposed by Hill and Middleton [57] predicts that in the thick periphyton matrix, surface algal cells are closer to the new (in our case ^13^C-labeled) inorganic carbon source, and it takes time for ^13^C labeled molecules to reach the deeper layers. Grazers, such as caddisflies, are proposed to be efficient in improving algal access to the new inorganic carbon because they are able to modify the matrix by feeding on deep periphyton layers [57]. However, this was most probably not the case in our laboratory experiment, where periphyton was formed by loosely attached diatoms that are unlikely to restrict matrix permeability for inorganic carbon and nutrients. In these incubations, the grazer-induced enhancement of ^13^C uptake per biomass unit was most likely due to stimulation of cell renewal and maintaining algal communities at early successional stages with high growth rates [58].

### C:N:P stoichiometry and carbon transfer efficiency

Many studies have demonstrated grazer-induced decrease in algal C:nutrient ratios in benthic algae due to nutrient recycling or increased nutrient availability and because of generally higher proportions of algae in relation to detritus (high C:nutrient ratios) in grazed substrates [38], [59], [32], [60], [61]. Our design of the laboratory experiment allows teasing apart the effects of physical disturbance and related structural changes in the periphyton community from the effects of nutrient recycling. This was possible because nutrients released by grazers were available for all periphyton in both grazed and non-grazed treatments, whereas direct contact between the grazers and the periphyton occurred only in the grazed treatments (Figure 2). Reductions in C:nutrient ratios in the grazed treatments should thus be attributed to physical disturbance, such as the reduction of periphyton mat thickness and the relative increase of nutrient availability, the removal of detritus and keeping the algal community at an early successional stage. Moreover, the overall increase in periphyton phosphorus content throughout the experiment, at least to some extent, is likely to have been induced by the biological stoichiometry of the grazers, as nitrogen-rich caddisflies are known to retain less phosphorus and thus increase P availability for benthic algae [59]. Thus, the caddisflies would improve food quality for other grazers by lowering C:P and C:N ratios in periphyton. However, the stoichiometric food quality increase along with the reduction in food quantity makes predictions about the net effects on consumer nutrition and growth rather difficult [59], [62].

In our study, we did not observe any pronounced difference in the C:nutrient ratio between the light and nutrient treatments. However, the carbon transfer efficiency from primary to secondary producers was significantly affected by light. In contrast, in pelagic systems the opposite effect has been reported, with low carbon transfer efficiency under high light and related increases in C:nutrient ratios [20]. These effects were, however, estimated at full daylight and at a 90% reduction of light, whereas our study involves low light levels typical for boreal forest humic streams, at which algae adapt by increasing their nutrient cell quota for photoadaptation at low light levels [23], [11]. Another important aspect in our experiment is the seemingly high heterotrophic production as indicated by the increase in P and AFDW and the relatively high ^13^C uptake attributable to bacteria and other heterotrophs as well as the energy loss through microbial respiration. As a result, the observed low energy transfer efficiency in such mixed communities under extremely shaded conditions may be expected when compared to autotrophic food chains under high light conditions. Also, there were distinct differences in algal community composition, with diatom dominance at low light and filamentous green algae at high light, which may have contributed to the observed differences in energy transfer efficiency. Although diatoms are often a preferable food source for invertebrate grazers [25], [50], a significant proportion of diatom cells can pass the guts of caddisflies undigested [63] and be consumed repeatedly [64], which implies a decrease in trophic transfer efficiency. By contrast, green algae at early stages of their growth might have served as a highly palatable food source, which enhances trophic transfer efficiency.

## Conclusions

In conclusion, the nutrient stoichiometry of periphyton dominated by diatoms and possessing high heterotrophic microbial activity did not follow predictions of the light:nutrient hypothesis [26], in which the C:nutrient ratio in the produced organic matter increase with increasing light, especially at low nutrient concentrations [29]. The predicted effect of high light and low nutrients is more likely to occur for algal communities dominated by opportunistic fast growing species, such as filamentous green algae; such communities are likely to develop following stream canopy removal [17], [55]. At early succession stages, these forms would represent a high quality food source for grazers and stimulate their growth as was shown by the enhanced ^13^C uptake in caddisflies. These findings suggest that at sub-saturating forest light levels, even a small increase in light intensity could result in community-wide effects on periphyton by changing the community dominance from diatoms to more diverse species composition, with different architecture of mats, and a subsequent increase in energy uptake and transfer in the food webs.

## Supporting Information

Figure S1 **Field experiment incubations in the open (A) and forested (B) stream sections at high flow and substrate sampling site for laboratory experiment at low flow conditions (C).**
(TIF)

Table S1 **Temporal variation of physicochemical parameters.**
(DOCX)

Table S2 **Data obtained in the field experiment.**
(XLSX)

Table S3 **Algal biovolume (10^6^ µm^3 ^cm^−2^).**
(XLSX)

Table S4 **Data obtained in the laboratory experiment.**
(XLSX)

Table S5 **Effect sizes of nutrient additions, light and grazing on periphyton biomass.**
(DOCX)
