# Supplementary figures and images for: Light Increases Energy Transfer Efficiency in a Boreal Stream
Source: PLoS One. 2014 Nov 20;9(11):e113675. doi: 10.1371/journal.pone.0113675 (PMC4239105; doi:10.1371/journal.pone.0113675)

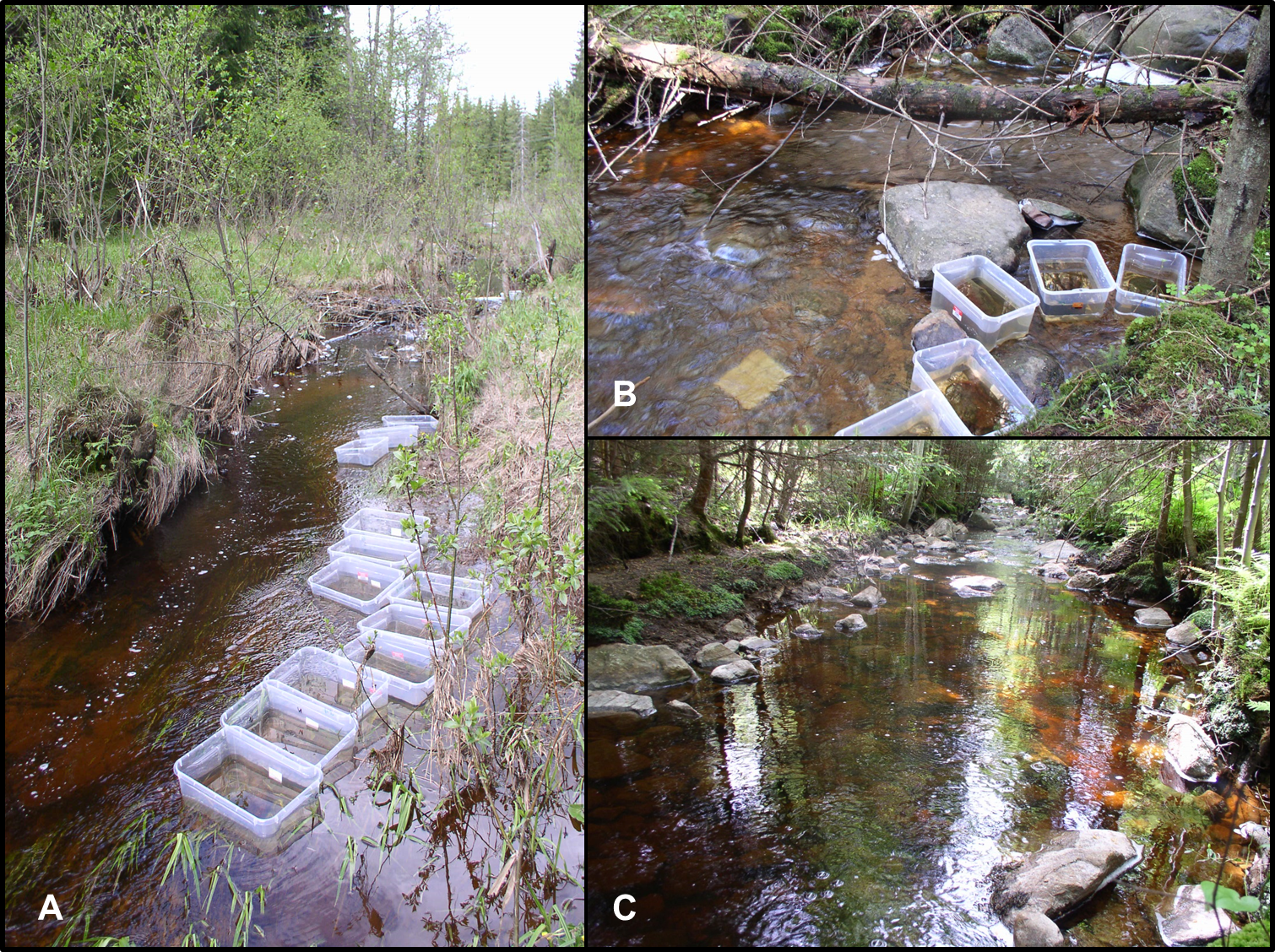

Supplement: Figure S1 — Field experiment incubations in the open (A) and forested (B) stream sections at high flow and substrate sampling site for laboratory experiment at low flow conditions (C). (TIF) [file pone.0113675.s001.tif]
