# Supplementary material for: Light Increases Energy Transfer Efficiency in a Boreal Stream
Source: PLoS One. 2014 Nov 20;9(11):e113675. doi: 10.1371/journal.pone.0113675 (PMC4239105; doi:10.1371/journal.pone.0113675)
Supplement: Table S1 — Temporal variation of physicochemical parameters. (DOCX) [file pone.0113675.s002.docx]

PLoS One │ Supporting Information
**Light increases energy transfer efficiency in a boreal stream**
Jūratė Lesutienė, Elena Gorokhova, Daiva Stankevičienė, Eva Bergman and Larry Greenberg

Table S1. Temporal variation of physicochemical parameters (temperature, photosynthetically active radiation (PAR), pH, conductivity, absorbance (at 455 nm wave length)) in the water during the field and laboratory experiments (mean ± SD).

|  | Time of the measurement | T, °C | PAR, μmol m^-2^ s^-2^ | | pH | Conductivity, μs cm^-1^ | Absorbance |
| --- | --- | --- | --- | --- | --- | --- | --- |
|  |  |  | LL | HL |  |  |  |
| *In situ* experiment | 19:00 | 10.1 ± 0.7 | 7 ± 2 | 78 ± 7 | 5.75 ± 0.03 | 23.1 ± 0.8 | 0.12 |
|  | 11:00 | 9.5 ± 0.7 | 33 ± 15 | 377 ± 419 | 5.80 ± 0.03 | 22.9 ± 0.8 | - |
|  | 14:00 | 12.0 ± 1.0 | - | 1075 | - | - | - |
| Laboratory experiment | Start | 14 | 6 ± 2 | 40 ± 4 | 6.3 | 31.6 | 0.16 |
|  | Day 2 | 14 | 6 ± 2 | 40 ± 4 | 6.9 ± 0.7 | 36.9 ± 6.6 | - |
|  | Day 5 | 14 | 6 ± 2 | 40 ± 4 | 7.4 ± 0.1 | 38 ± 7.7 | 0.15 ± 0.01 |
|  | Day 14 | 14 | 6 ± 2 | 40 ± 4 | 7.6 ± 0.4 | 37.5 ± 7.9 | 0.13 ± 0.01 |
