# Supplementary material for: Light Increases Energy Transfer Efficiency in a Boreal Stream
Source: PLoS One. 2014 Nov 20;9(11):e113675. doi: 10.1371/journal.pone.0113675 (PMC4239105; doi:10.1371/journal.pone.0113675)
Supplement: Table S5 — Effect sizes of nutrient additions, light and grazing on periphyton biomass. (DOCX) [file pone.0113675.s006.docx]

PLoS One │ Supporting Information
**Light increases energy transfer efficiency in a boreal stream**
Jūratė Lesutienė, Elena Gorokhova, Daiva Stankevičienė, Eva Bergman and Larry Greenberg

**Table S5. Effect sizes of nutrient additions, light and grazing on periphyton biomass** (chlorophyll *a* and AFDW), inorganic carbon uptake (**Δ**^13^C, biomass-specific ^13^C uptake (Δ^13^C/Chl *a* and Δ^13^C/AFDW), nutrient content (C, N, and P) and nutrient ratios (N:P, C:P, and C:N) after 2, 5 and 14 days of laboratory experiment, repeated measures ANOVA. Numbers represent effect size η^2^. Significant effects are in bold: *p<0.05, **p<0.01, ***p<0.001; for marginally significant effects, p values are given. The 3rd order interactions are not presented as none were significant.

| **Parameter** | **Chl *a*** | **AFDW** | **Δ^13^C** | **Δ^13^C/Chl *a*** | **Δ^13^C/AFDW** | **C** | **N** | **P** | **N:P** | **C:P** | **C:N** |
| --- | --- | --- | --- | --- | --- | --- | --- | --- | --- | --- | --- |
| **After 2 days** |  |  |  |  |  |  |  |  |  |  |  |
| Light | 0.08 | 0.01 | **0.47*** | 0.12 | 0.07 | 0.00 | 0.02 | 0.03 | 0.00 | 0.01 | 0.12 |
| Nutrients | 0.06 | 0.10 | 0.06 | 0.13 | 0.15 | 0.18 | 0.18 | 0.17 | 0.01 | 0.02 | 0.01 |
| Light×nutrients | 0.00 | 0.01 | 0.14 | 0.00 | 0.00 | 0.00 | 0.04 | 0.03 | 0.21 | 0.08 | 0.36^0.07^ |
| Grazing | **0.43*** | **0.59**** | 0.01 | 0.25 | **0.44*** | **0.49*** | 0.29 | 0.23 | 0.26 | 0.12 | 0.35^0.07^ |
| Grazing×light | 0.01 | 0.00 | 0.07 | 0.04 | 0.04 | 0.04 | 0.04 | 0.09 | 0.11 | 0.08 | 0.02 |
| Grazing×nutrients | 0.01 | 0.03 | 0.12 | 0.02 | 0.04 | 0.00 | 0.00 | 0.00 | 0.00 | 0.00 | 0.06 |
| **After 5 days** |  |  |  |  |  |  |  |  |  |  |  |
| Light | 0.06 | 0.03 | **0.57*** | 0.36^0.07^ | 0.24 | 0.01 | 0.00 | 0.01 | 0.01 | 0.03 | 0.00 |
| Nutrients | 0.02 | 0.00 | 0.01 | 0.01 | 0.01 | 0.00 | 0.02 | 0.02 | 0.00 | 0.00 | 0.06 |
| Light×nutrients | 0.01 | 0.00 | 0.02 | 0.00 | 0.01 | 0.00 | 0.00 | 0.03 | 0.01 | 0.04 | 0.05 |
| Grazing | **0.88***** | **0.86***** | **0.53*** | **0.82***** | **0.88***** | **0.89***** | **0.89***** | **0.82***** | 0.06 | **0.53*** | **0.47**^*^ |
| Grazing×light | 0.22 | 0.36^0.07^ | 0.02 | 0.28 | **0.41*** | 0.18 | 0.24 | 0.13 | 0.02 | 0.02 | 0.01 |
| Grazing×nutrients | 0.00 | 0.02 | 0.05 | 0.00 | 0.00 | 0.00 | 0.02 | 0.00 | 0.13 | 0.04 | 0.07 |
| **After 14 days** |  |  |  |  |  |  |  |  |  |  |  |
| Light | 0.00 | 0.00 | **0.67**** | **0.41*** | 0.37^0.06^ | **0.51*** | 0.17 | 0.02 | 0.21 | 0.29 | 0.11 |
| Nutrients | 0.15 | 0.00 | 0.02 | 0.11 | 0.12 | 0.03 | 0.05 | 0.18 | 0.03 | 0.04 | 0.03 |
| Light×nutrients | 0.16 | 0.07 | 0.01 | 0.02 | 0.05 | 0.11 | 0.06 | 0.04 | 0.06 | 0.07 | 0.00 |
| Grazing | **0.83***** | **0.72**** | **0.64**** | **0.81***** | **0.83***** | **0.90***** | **0.90***** | 0.29 | **0.67**** | **0.77***** | **0.65**** |
| Grazing×light | 0.00 | 0.21 | 0.04 | 0.02 | 0.09 | 0.34 | 0.27 | 0.04 | **0.53*** | **0.57*** | 0.18 |
| Grazing×nutrients | 0.07 | 0.00 | 0.00 | 0.00 | 0.00 | 0.12 | 0.16 | 0.00 | 0.23 | 0.20 | 0.01 |
